# The whistle-smile reflex in Parkinson's disease: an exploratory case-control study

**DOI:** 10.1055/s-0046-1829023

**Published:** 2026-10-23

**Authors:** Mateus Damiani Monteiro, Eurípedes Gomes de Carvalho Neto, Liana Lisboa Fernandez, Arlete Hilbig, Carlos Roberto de Mello Rieder

**Affiliations:** 1 Universidade Federal de Ciências da Saúde de Porto Alegre, Departamento de Neurologia, Porto Alegre RS, Brazil.

**Keywords:** Parkinson Disease, Neurological Examination, Neurologic Manifestations

## Abstract

**Background:**

Parkinson's disease (PD) is a well-known neurodegenerative disease characterized by tremors, rigidity, and bradykinesia. In 1943, Frederic Hanes described the whistle-smile reflex (WSR), noting its absence in PD patients due to reduced facial movements.

**Objective:**

To assess the WSR's diagnostic performance.

**Methods:**

In the current cross-sectional study, we selected PD patients and healthy controls. They were filmed while asked to whistle, and a blinded physician assessed if the individuals smiled or not. Absence of smiling after whistling was considered a positive test. We stratified the smiling intensity into 4 groups. We calculated the odds ratio (OR) of the positive WSR in PD and asymptomatic patients, which was reported with CIs, sensitivity (SE), specificity (SP), positive predictive value (PPV), negative predictive value (NPV), and we plotted a receiver operating characteristic (ROC) curve.

**Results:**

We identified 43 patients with PD (mean age = 63 ± 10 years; 30% of female subjects) and 44 asymptomatic controls (mean age = 64 ± 10 years; 51% of female subjects). The WSR test was positive in 35/43 PD cases versus 7/44 in the asymptomatic group (OR = 23.1; 95%CI = 7.6–70.5; SE = 81.4%; SP = 84.1%; PPV = 83.3%; NPV = 82.2%). When stratified considering the group with the least intense smile as a positive test, the test was positive in 42/43 PD cases versus 23/44 in the asymptomatic group (OR = 38.3; 95%CI = 4.8–303.7; SE = 97.7%; SP = 47.7%; PPV = 64.6%; NPV = 95.4%).

**Conclusion:**

The WSR test showed a good performance in identifying PD patients; it may complement the physical examination, and its result may be a useful finding during the assessment of a patient with PD.

## INTRODUCTION

Parkinson's disease (PD) is a well-known neurodegenerative condition caused by the impairment of the encephalic dopaminergic system. 1 Its cardinal signs and symptoms include bradykinesia, rigidity, and tremor. Impaired balance, constipation, hyposmia, rapid eye movement (REM) sleep behavior disorder, and lack of facial expression are also prominent features. 2 3

The diagnosis of parkinsonism is made clinically with the presence of bradykinesia plus rigidity and/or tremors. 4 In 1943, Dr. Frederic Hanes 5 described the whistle-smile reflex (WSR), also known as *Hanes’ sign* : while asked to whistle, a healthy person would smile after whistling (maybe due to the doctor's unusual request); while the patient with PD would not, due to facial hypomimia.

Forgotten across the years, the WSR was recently revisited, 6 but its applicability in the clinical practice remains unclear. 7 In the current study, we aim to assess the WSR as a reliable physical examination finding during the diagnosis of PD.

## METHODS

We prospectively selected PD patients and healthy controls (HCs) from a movement disorders outpatient clinic from October 2021 to March 2022. Patients with the diagnosis of PD according to the Movement Disorder Society (MDS) clinical diagnostic criteria for PD were eligible, and the HCs were selected from the patients’ asymptomatic relatives. We excluded patients with oral dyskinesias, as well as those with a previous diagnosis of depression or another psychiatric comorbidity. They were individually recorded on **Video 1** while asked to whistle. The command was given in a neutral tone, and no one else was allowed to stay in the room during the filming to avoid interference during the procedure.

**Video 1:** Examples of the WSR. The reflex is absent in the first segment, while the following person has the reflex present (available from: https://www.arquivosdeneuropsiquiatria.org/wp-content/uploads/2026/07/ANP-2026.0006-Video.mp4 ).

The Hoehn and Yahr scale, on-off status, previous psychiatric diagnosis, medication in use, and time since the last intake were obtained during the **Video 1** recording. The on-off status was assessed by the attending neurologist during the clinical evaluation. The duration of the disease was obtained later via the electronic medical record.

The videos were assessed by an independent blinded non-neurologist physician who was asked about the presence or absence of the smile before, during, and/or after the whistling and the degree of the smile. The images in **Video 1** were neither edited nor cropped. We adapted a previously-published smile taxonomy 8 into a smiling scale spanning 4 stages, from a simple corner-of-the mouth movement to a big laugh ( Figure 1 ). The rater was instructed to classify the patients’ smiles according to the visual scale, divided into a small smile without mouth opening, a smile showing teeth but no other significant facial muscle contraction, a big smile with involvement of upper face muscle contraction, and a laugh.

**Figure 1:**
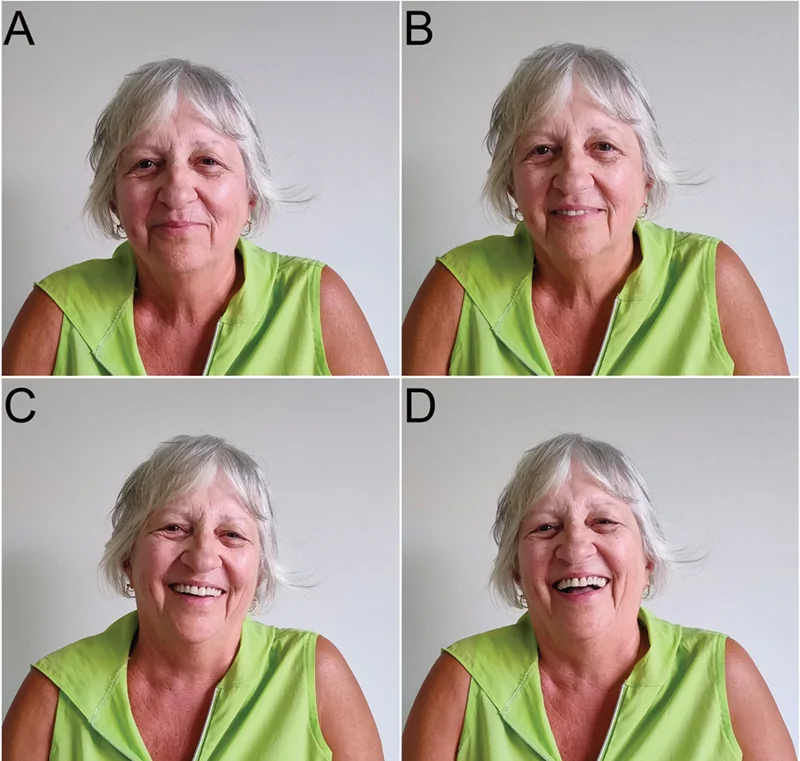
Samples of smiling intensity. From degrees 1 ( **A** ) to 4 ( **D** ).

We did not use a neurologist to minimize the bias of knowing the diagnosis of the participants by the eventual recognition of the masked face. The Hanes’ sign was considered present—or the WSR was negative, and the test, positive—when there was not a smile before, during, and/or after whistling. It was considered for evaluation even if the whistle was incomplete or the patient was unable to do it but made a genuine attempt.

Since the present was an experimental study about spontaneous smiling in hypokinetic patients and considering that some of those patients might still have enough facial movement for a small smile, we decided to sensitize the test considering the grade-1 smile (corner-of-the-mouth smile) as a positive test.

We calculated the odds ratio (OR) of the sign in the PD patients and HCs, which was reported along with CIs, sensitivity (SE), specificity (SP), positive predictive value (PPV), and negative predictive value (NPV), and we plotted a receiver operating characteristic (ROC) curve using the degrees of the smiles as thresholds. We used binary logistic regression to assess the association of the WSR with disease duration and the Hoehn and Yahr scale, and the Chi-squared (χ ^2^ ) test, to assess the association between WSR and the on-off status. The statistical analysis was performed with the IBM SPSS Statistics for windows (IBM Corp.) software, version 27.0.

The current study was approved by the Ethics Committee of Irmandade da Santa Casa de Misericórdia de Porto Alegre (under number 54555421.3.0000.5335, on February 3rd, 2022), and it followed the Strengthening the Reporting of Observational Studies in Epidemiology (STROBE) guidelines for observational studies. Signed informed consent was obtained from all participants.

## RESULTS

We enrolled 43 PD patients and 44 HCs; there was no statistically significant difference in terms of age between the groups ( *p* = 0.81), but there was a difference in the frequency of patients of the female sex ( *p* = 0.007). No difference was found in the WRS prevalence between male and female subjects within the PD patients ( *p* = 0.62) or HCs ( *p* = 0.909). Among the PD patients, the median duration of the disease was of 8 (5–13) years, the median Hoehn and Yahr ( *n* = 42) stage was 2.25 (2–2.5), and 32 patients were in the on status. All patients were on levodopa therapy, and the median time since the last levodopa intake was of 2.5 (1.5–3.5) hours. In total, 23 patients were also taking pramipexole, and the median time since the last intake was of 3.5 (2.5–10.25) hours. There was missing information from 1 patient regarding the Hoehn and Yahr scale ( Table 1 ).

**Table 1:** Demographics of the study sample

	Parkinson's disease ( *n* = 43)	Healthy controls ( *n* = 44)	*p* -value
Female sex: n (%)	13 (30.2%)	26 (59.1%)	0.007
Mean age (years)	64 ± 10	63 ± 10	0.81
Mean disease duration (years)	8 [5–13]		
Median score on the Hoehn and Yahr scale ( *n* = 42)	2.25 (2–2.5)		
On status: n (%)	32 (74.4%)		
Median time since last levodopa intake (hours)	2.5 (1.5–3.5)		
Median time since last pramipexole intake (hours)	3.5 (2.5–10.25)		

The WSR was absent (positive test result) in 35/43 PD patients and only in 7/44 HCs (OR = 23.1; 95%CI = 7.6–70.5; *p* < 0.001). The SE, SP, PPV, and NPV were of 81.4% (95%CI = 67.7–90.9), 84.1%, (95%CI = 70,9–92,1), 83.3% 95%CI = 69,6–91,3), and 82.2% 95%CI = 68.0–91,0) respectively.

Alfer smile stratification and considering grade-1 smiles (just a discrete corner-of-the-mouth movement) a non-smiling response, the test was positive in 42/43 PD patients and in 23/44 HCs (OR = 38.3; 95%CI: 4.8–303.7; *p* = 0.001). The SE, SP, PPV, and NPV were of 97.7% (95%CI = 88.7–99.6) 47.7% (95%CI = 33.5–62.3), 64.6% (95%CI = 52,5–75.0), and 95.5% (95%CI = 78.6–99.2) respectively ( Table 2 ). The ROC curve showed an area under the curve (AUC) of 0.859 (95%CI = 0.777–0.941) ( Figure 2 ).

**Table 2:** Test results of the study sample

	Whistle-smile reflex	Sensitized test
	
Odds ratio (95%CI)	23.1 (7.6–70.5; *p* < 0.001)	38.3 (4.8–303.7; *p* = 0.001)
Sensitivity (95%CI)	81.4% (67.7–90.9%)	97.7% (88.7–99.6%)
Specificity (95%CI)	84.1% (70.9–92.1%)	47.7% (33.5–62.3%)
Positive predictive value (95%CI)	83.3% (69.6–91.3%)	64.6% (52.5–75.0%)
Negative predictive value (95%CI)	82.2% (68.0–91.0%)	95.5% (78.6–99.2%)

**Figure 2:**
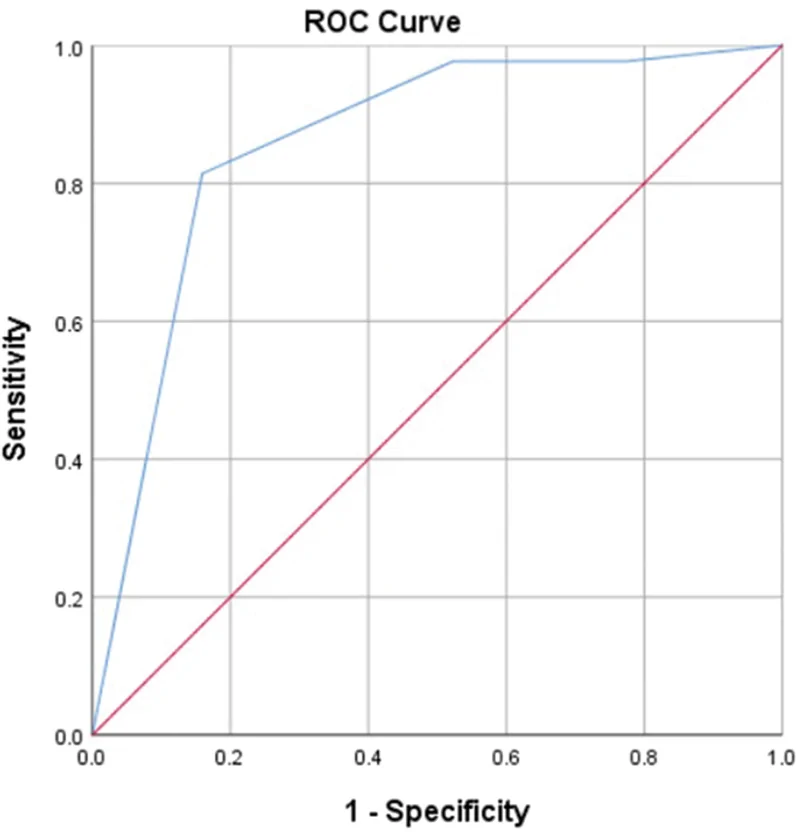
Receiver operating characteristic (ROC) curve showing the performance on the whistle-smile reflex (WSR) test. The area under the curve represents the ability to differentiate between Parkinson's disease (PD) patients from healthy controls (HCs).

We found no association between the test result and disease duration (OR = 0.97; 95%CI = 0.83–1.12; *p* = 0.740), the Hoehn and Yahr scale (OR = 0.72; 95%CI = 0.23–2.27; *p* = 0.579), nor the on-off status (χ ^2^ = 0.733; *p* = 0.392). Although one of four cells (25%) had an expected count lower than 5, the result of the χ ^2^ test was consistent with that of the Fisher's exact test ( *p* = 0.401) ( Table 3 ).

**Table 3:** Clinical associations found in the study

	Whistle-smile reflex		*p* -value
	Present	Absent	

On status: n (%)	5 (15.6)	27 (84.4)	0.392
Off status: n (%)	3 (27.3)	8 (72.7)	
			
Disease duration: odds ratio (95%CI)	0.97 (0.83–1.12)		0.740
Hoehn and Yahr scale: odds ratio (95%CI)	0.72 (0.23–2.27)		0.579

To address the sex difference between the groups, we performed a binary logistic regression test including sex and age. The association of the WSR with PD remained statistically significant (OR = 21.6; 95%CI = 6.9–67.8; *p* < 0.001).

## DISCUSSION

We found the WSR to have good SE and SP, with both rates above 80%, an increase compared with previously-reported rates. 7 When sensitized, the NPV reaches 95.4%.

The prevalence of PD in the population of the current study is significantly greater than in the general population, and the PPV and NPV vary according to the prevalence of the disease. The NPV is inversely proportional to the prevalence of the disease, making a smile bigger than a small corner-of-the-mouth movement virtually excluding PD.

Facial akinesia can be present 9.8 years before the diagnosis of PD, particularly in individuals with REM sleep behavior disorder. 9 This highlights the importance of testing the WSR in this population as a potential preclinical marker of PD, enabling early treatment. Moreover, the possibility of the emergence of a disease-modifying drug 10 makes the early diagnosis crucial.

One of the study limitations is the small number of participants, although it is the biggest study about the WSR to date. Although the patients were not stratified according to the Hoehn and Yahr scale, or considering if they were “on” or “off,” we looked at those clinical variables and found no evidence of association of the presence/absence of the WSR with them.

Even a non-neurologist can identify characteristics suggestive of PD, leading to a limitation regarding blinding. To minimize this limitation, the rater was only asked to identify the smile and grade it. The rater had no access to any other aspect of the methodology and is not an author of the study.

Although we did not assess the inter-rater agreement, it had already been demonstrated to have good reliability among raters. 7 This result is not necessarily applicable to non-neurologists and having only one rater in the present study is also a limitation.

The median disease duration was 8.47 years. At this point, the PD manifestations are exuberant, and a subtle sign would lose its value. However, binary logistic regression showed that the duration of the disease has no influence on the presence/absence of the WSR, demonstrating that the sign could be useful even in the early stages of the disease.

There was a frequency difference in terms of female subjects between the PD and HC groups, which can lead to a selection bias. However, the logistic regression analysis demonstrated that PD remains independently associated with a positive test.

The WSR showed promising discriminatory performance in the present exploratory case-control study. Larger blinded studies including early and prodromal PD populations are needed before routine clinical use can be recommended.
